# Comparative Analysis of the Selected Photoreceiver Input Stages in Terms of Noise

**DOI:** 10.3390/s25051359

**Published:** 2025-02-23

**Authors:** Krzysztof Achtenberg, Zbigniew Bielecki

**Affiliations:** Institute of Optoelectronics, Military University of Technology, 2 Kaliskiego Str., 00-908 Warsaw, Poland; zbigniew.bielecki@wat.edu.pl

**Keywords:** noise, photodiode, TIA, transimpedance amplifier, radiation detection

## Abstract

Semiconductor radiation detectors usually use a specific signal conditioning circuit, ensuring the required detection system parameters. This paper details the noise properties of specific input stages in photoreceivers that detect various types of radiation. For this purpose, the popular silicon PIN photodiode (BPW34) and two different types of low-noise operational amplifiers (AD797A and ADA4625-1) were used. In the presented experiments, noise measurements were provided for voltage and transimpedance amplifiers operating in input stages, comparing their noise and bandwidths. This made it possible to obtain results for bipolar junction transistor (BJT)- and field-effect transistor (FET)-based input stages of circuity, cooperating directly with a photodiode. Analyzing the obtained characteristics and considering the photodiode operation mode, it is evident that the transimpedance amplifier and photoconductive mode should be considered a typical first-choice solution. In some cases, the performances, such as bandwidth and noise, may be similar to those of voltage. Nevertheless, the bias method used in TIA and feedback compensation can also affect the resulting output noise spectral characteristics due to the photodiode and other capacitances existing in the circuit. In the case of a high transimpedance, the FET-based op-amps ensure lower output noise than the BJT-based ones due to the significantly lower current noise. The simple radiation detector with two-channel differential TIA was also proposed and tested based on the results obtained.

## 1. Introduction

Photoreceivers play a significant role in a wide range of radiation detection methods. It can be found in many applications, such as medicine, science, industry, and security systems. Such devices determine radiation intensity, time-of-light, particle counting, and other measurements [1,2]. The recent intense work in this area caused different devices and techniques to be invented and developed [3]. In many cases, solid-state detectors are used. Analyzing a few recent works, studies of organic and perovskite X-ray detectors, flexible or plastic scintillators, and Ga_2_O_3_ can be found [4,5,6,7,8].

The reverse-biased p-n junction of a diode is commonly used for radiation detection [9]. In this case, a wide depletion region, reduced dark current, and lower capacitance are obtained as benefits. Moreover, the different types of detectors (i.e., p-n, avalanche, or PIN photodiodes) where semiconductor junctions exist can be used [10,11]. The constructed devices can use a single pixel or a matrix of detectors. Solid-state photomultipliers (SSPMs) coupled with scintillator material have become very popular in dosimetry due to their high quantum efficiency, low dimensions, and low supply voltage compared to classic photomultipliers (PMTs). Metal oxide semiconductor field effect transistors (MOSFETs), static random-access memory (SRAM), and 3D NAND flash devices can also be used to construct specific radiation detectors [12]. Nevertheless, in most cases, proper signal conditioning must be ensured to prepare a signal that can be appropriately encoded by the subsequent processing circuit (i.e., analog-to-digital converter or comparator).

In this paper, we presented the noise performances of different input stages operating with silicon-based photodiodes. The results obtained can be used to select the proper circuit design approach for specific detection applications and requirements. Moreover, the two-channel differential TIA was also proposed to operate as a radiation detector with a PIN photodiode. The benefits obtained from two differential channels were also discussed.

The input stage of an optical radiation photoreceiver consists of a photodetector and amplifier. The photodetector converts the incident optical radiation into an electrical signal before amplifying it for further processing to extract useful information from the optical signal. The implemented amplifier in the detector circuit must ensure a low noise level and a sufficiently wide bandwidth to undistort the shape of the input waveforms [13]. Designing a low-noise photoreceiver system is a non-trivial task [14]. It is required to minimize the noise contribution from various sources such as background noise, photodetector leakage current, the thermal noise of biasing resistors, supply, and used semiconductor active elements (discrete transistors, op-amps) [15]. The latter can make the most important contribution apart from the non-reducible noise generated in the connected photodetector itself. The few recent works showing the results of dedicated ASIC preparation are presented in [16,17,18]. Nevertheless, the works focused on circuits created from available discrete elements can also be found in the recent literature [19,20,21].

**Figure 1:**
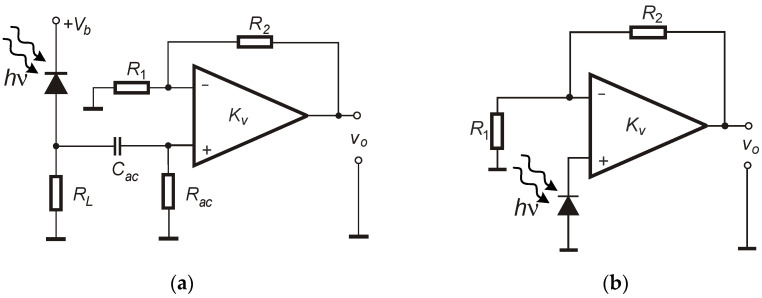
Schematic diagram of the first stage of the photoreceiver (**a**) with a voltage amplifier and (**b**) the amplifier allowing the photodiode to work in the fourth quarter of the characteristics *I*-*V*—photovoltaic mode.

Amplifiers used in the construction of photodiode-based detector front-end electronics can be divided into a few main groups, namely voltage, differential, current–voltage converters, and integrators (charge amplifiers) [22,23]. Voltage amplifiers (Figure 1) operating with photodiodes do not provide good linearity and dynamics of signal changes regarding the incident optical power. Moreover, in some cases, the frequency bandwidth of operation is also significantly limited. Current-to-voltage converters (transimpedance amplifiers—TIA) are commonly used with single-pixel visible and infrared radiation detectors, whereas charge amplifiers usually operate as readout circuits in detector arrays [24].

This paper will consider voltage and transimpedance amplifiers in terms of their noise properties and bandwidth during operation with a typical silicon-based PIN photodiode [25]. The obtained characteristics will be supplemented by proper theoretical-based discussions.

Now, let us consider voltage amplifier circuits for photodiodes. Depending on the type of detector and its specific applications, it can be biased from the source *V_b_* or not by simply connecting to the circuit ground potential. By preparing a specific configuration, the photodiode can operate in a photoconductive (Figure 1a) or photovoltaic mode (Figure 1b). In the first case, the photodiode is supplied from a low-noise DC voltage source and is loaded with the resistance *R_L_* (creating voltage divider). Incident radiation generates a photocurrent flowing through *R*_L_ and causes changes in a voltage drop at this resistance. To obtain high gain, the AC coupling must be applied to not saturate the op-amp output by the DC component, so this input stage can be used only with variable signals. In contrast, no voltage source supplies the photodiode in the second case (Figure 1b). In this case, the voltage generated by the photodiode is amplified by a non-inverting configuration with an op-amp. In general, this circuit is not recommended and is essentially unused because of the nonlinearity of voltage generated by the photodiode, especially in the case of strong radiation power. The advantages of reverse-biasing photodiodes include reducing their junction capacitance and increasing photoreceiver bandwidth. Due to this fact, datasheets of some photodiodes often present the dependence of photodiode capacitance on the applied reverse voltage [26].

**Figure 2:**
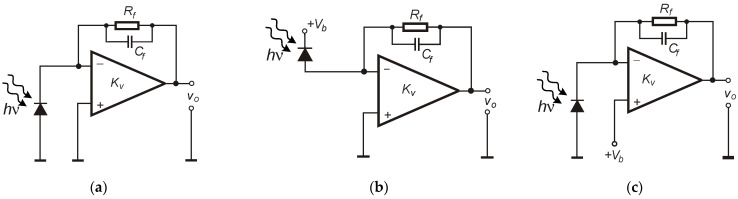
Photodiode operation (**a**) with an *I*-*V* converter without biasing—photovoltaic mode (**b**), (**c**) with reverse biasing—photoconductive mode.

Figure 2 shows several variants of photodiode operation with a current–voltage converter using an op-amp. The transimpedance is set by the feedback resistance value (*R_f_*). In all cases, the photodiode is connected to the inverting input (*IN*−). This can be performed using a cathode or anode, but it must be remembered that it affects output signal polarity. The symmetrical dual power supply of the op-amp is recommended. Nevertheless, the single supply is also possible and often used in many applications, but the low-noise reference voltage must be applied to the non-inverting node (*IN*+). The photodiode can be unbiased (Figure 2a) or biased using different approaches (Figure 2b,c). The negative bias voltage can also be used, but it must be applied to the photodiode anode. In the case of providing bias using non-inverting input (Figure 2c), it must be remembered that the maximum voltage produced across the photodiode depends on op-amp supply conditions and the relation between the photodiode and feedback resistance. Thus, the output dynamic range can be limited in some cases due to the relatively high DC component at the op-amp output node. The proper bias voltage across the photodiode will equal the *V_b_* when *R_d_* >> *R_f_* and op-amp operate in a dynamic range. In practice, for many cases, significant reverse biasing (i.e., >10 V) using a non-inverting node requires an op-amp that can be supplied by a relatively high voltage (and also can produce the required voltage at its output).

In general, the presented circuits can operate with various types of signals. Nevertheless, for specific applications, the key parameters can be significant. To operate with short pulsed signals, bandwidth limitation and appropriate amplifier frequency compensation should be considered so as to not to disturb the shape of the incident radiation signal (amplitude, slopes). Biased PD (with small capacitance) is usually used with TIA configuration in high-speed applications. The high-pass filter in voltage configuration (Figure 1a) can also affect the applied signal, especially at low frequencies. Gain reduction may be necessary to ensure operation within the amplifier’s dynamic range for high-power incident radiation signals.

**Figure 3:**
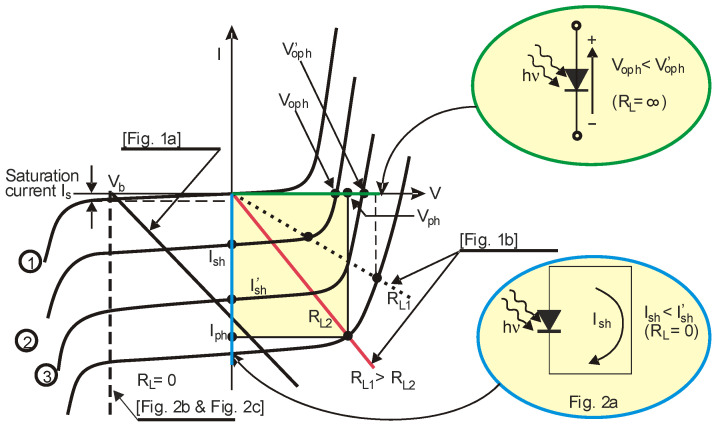
Current–voltage characteristics of photodiodes with load lines (adopted after [27]).

To understand the differences in the specific photodiode operation in these circuits, let us consider the current vs. voltage characteristics (*I*-*V*) of the photodiodes illuminated and unilluminated. Figure 3 shows typical *I*-*V* and “load” lines for a silicon-based photodiode operating in a photovoltaic and photoconductive mode. The photodiode operating point results from the intersection of the specific load line with the *I*-*V* characteristics.

When the photodiode operates in a photoconductive mode (Figure 1a), the load line (black line in Figure 3) is determined by the photodiode bias voltage *V_b_* and the load resistance value *R_L_*. Suppose the photodiode is reverse-biased with the voltage *V_b_*; even without illumination, some dark current flows exist—*I_dark_*—which is the shot noise source. An increase in illuminance causes an increase in the *I_ph_* current. In this case, the total current is equal to the sum of the photocurrent, the background current, and the dark current.

(1) $$I_{d}=I_{\mathrm{ph}}+I_{\mathrm{bg}}+I_{\mathrm{dark}}=R_{I}AE_{e}+I_{\mathrm{bg}}+I_{\mathrm{dark}},$$

where *R*_I_ is the photodiode’s current responsivity, *A* is the detector area, and *E*_e_ is the irradiance.

Figure 1b presents a simplified schematic of an amplifier that enables the photodiode to operate in the fourth quadrant of the *I*-*V* characteristics (photovoltaic mode). When the input impedance of the operational amplifier is significantly higher than the photodiode’s shunt resistance (*R_in_* >> *R_sh_*), the system meets the condition for an “open circuit” operation. The equation defines the voltage at the preamplifier output.

(2) $$V_{o}=\frac{R_{1}+R_{2}}{R_{1}}\frac{\mathrm{kT}}{q}\ln\left[\frac{I_{\mathrm{ph}}+I_{s}}{I_{s}}\right].$$

It varies logarithmically with the intensity of the incident radiation. However, a drawback of this solution is the exponential relationship between the saturation current *I_s_* and the detector temperature. Therefore, in many applications, the detector temperature must be precisely stabilized. The photovoltaic mode of operation is mainly used in solar cells when generating the required photovoltage in the maximum power point (MPP), which is the most important. In the case of this mode (Figure 1b), the load line passes through the origin of the coordinate system and intersects the photodiode characteristics in the fourth quadrant. When *R_L_* = ∞, the load line (green line in Figure 3) overlaps with the x-axis. At this point, the voltage *V_oph_* can be determined—this represents the voltage where the *I*-*V* characteristic intersects the x-axis, known as the open-circuit voltage *V_oph_*. This voltage exhibits a logarithmic dependence on variations in the incident radiation power.

For lower load resistance values, the photodiode operating point is determined by the voltage *V_ph_* and the photocurrent *I_ph_*. In this case, measuring the voltage *V*_ph_ and the current *I*_ph_ is possible. The value of the resistance *R_L_*, for which the product *V_ph_I_ph_* takes the maximum value (MPP), should be treated as the optimal load resistance *R_Lopt_* (red line in Figure 3). If *V_b_* is zero (Figure 2a), the load line aligns with the y-axis (blue line in Figure 3). In this case, the output current is directly proportional to the irradiance. This current, known as the photodiode short-circuit current, is given by the following equation:

(3) $$I_{\mathrm{sh}}=R_{I}AE_{e}.$$

In Equation (3), the dark current component can be omitted.

The transimpedance amplifier provides a load resistance close to zero (because of its low input resistance). The short-circuit current exhibits excellent linearity in response to changes in irradiance. This linearity is influenced by the type of photodiode and the circuit in which it operates. Satisfactory linearity can be achieved for variations in incident radiation power ranging from a few pW to mW.

Linearity is constrained at the lower end by the NEP value and at the upper end by the load resistance and supply voltage. The circuit configurations shown in Figure 2b,c retain the advantages of the setup in Figure 2a while also maintaining a constant photodiode bias voltage. An ideal amplifier’s voltage drop across the photodiode equals the bias voltage *V_b_*. Applying a reverse bias reduces the photodiode junction capacitance, thereby increasing the upper frequency limit of the photoreceiver input stage.

Another advantage of this circuit is a slight increase in the input signal dynamic range. To obtain a high gain for the *I*-*V* converter, it is necessary to use the high-value resistor *R_f_*. Along with this resistance increase, its thermal current noise is decreasing. It should be considered that the existing in the circuit parasitic capacities also increase the time constant and reduce the resulting bandwidth. In many applications, two or more stage amplifiers are used to obtain a wide bandwidth. For this purpose, the transimpedance of the first stage is usually reduced. The overall circuit transimpedance can be further increased by applying the following voltage stages.

**Figure 4:**
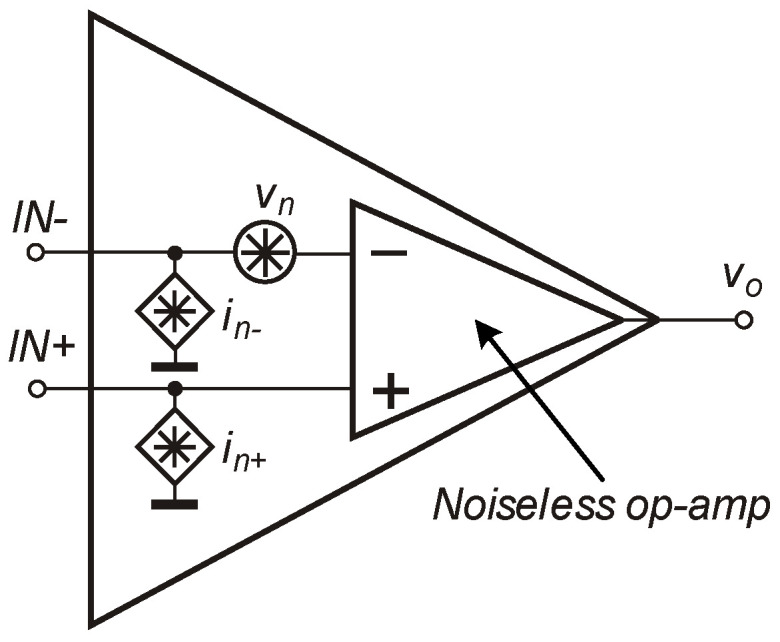
The noise model of an operational amplifier.

Considering the noise in the specific circuits, some information about the existing sources must be provided first. In Figure 4, a schematic diagram of the op-amp equivalent noise is presented. In such devices, we have equivalent input voltage noise sources (*v_n_*) and equivalent input current noise sources at both inputs (*i_n_*_−_ and *i_n+_*). The influence of these sources can depend on the resistances existing in the circuit, including signal source (photodiode) resistance or impedance. Moreover, it must also be remembered that each resistance is also a source of thermal noise (4*kTR*). The proper calculation, taking into consideration specific noise gain that in some cases is not equal to signal gain, led to obtaining noise referred to as output (RTO). In some cases, the input-referred value (RTI) is more preferable. The information about detailed noise considerations in voltage amplifiers can be found in many papers and books [28,29]. Noise description and estimation in transimpedance amplifier circuits can be found in [30].

In the case of a transimpedance amplifier, the total equivalent input noise is the sum of the noise mean squares from the photocurrent *I_ph_*, detector dark current *I_d_*, background current *I_b_*, thermal noise of the resistor *R_f_*, and amplifier noise sources, including current *I*_n_ and voltage *V*_n_. Thus, the input-referred current mean-squared noise will take the form of the following:

(4) $$I_{\mathrm{nTIA\_RTI}}^{2}=I_{\mathrm{nph}}^{2}+I_{\mathrm{ndark}}^{2}+I_{\mathrm{nbg}}^{2}+I_{n}^{2}+\frac{4\mathrm{kT}∆f}{R_{d}}+\frac{4\mathrm{kT}∆f}{R_{f}}+{\left(\frac{V_{n}}{R_{f}}\right)}^{2},$$

where *k* is the Stefan–Boltzmann constant and *R_d_* is the photodiode differential resistance. In Equation (4), we assumed that *R_d_* >> *R_f_*. To describe RMS noise at the output, Equation (5) can be used. In this simplified equation, we assumed the negligible influence of capacitances, grounded non-inverting input (IN+), and that *I_n_*_+_ = *I_n_*_−_ = *I_n_*:

(5) $$V_{\mathrm{nRTO\_TIA}}=\sqrt{{{\left(I_{\mathrm{nph}}R_{f}\right)}^{2}+\left(I_{\mathrm{ndark}}R_{f}\right)}^{2}+{\left(I_{\mathrm{nbg}}R_{f}\right)}^{2}+{\left(I_{n}R_{f}\right)}^{2}+\frac{4\mathrm{kT}∆f}{R_{d}}R_{f}^{2}+4\mathrm{kT}R_{f}∆f+{V_{n}}^{2}}.$$

In Equations (4) and (5), the RMS values of specific noise components calculated in the desired noise bandwidth (∆*f*) are used. If noises other than white occur, the integral operation in noise bandwidth must be used. Moreover, the result of the calculation can be used only in some cases, when input and feedback impedances can be simplified to resistances and a low-frequency range. In general, noise gain in TIA must be defined as 1 + *Z_f_*/*Z_in_* (*Z_f_*—feedback impedance, *Z_in_*—input network impedance). This indicates that the output noise spectral characteristic is formed to a non-trivial shape that can be divided into specific regions described by complex equations. Detailed analyses of this topic can be found in [31,32]. In the case of TIA, the photodiode capacitance can significantly impact output noise characteristics. The detailed consideration can be found in [21,33].

In a photoreceiver with a transimpedance amplifier, a similar frequency response can be achieved using a feedback resistor *R_f_* higher than the load resistor *R_L_* found in a photoreceiver with a voltage amplifier. Consequently, the transimpedance amplifier provides a higher signal-to-noise ratio (SNR) for the same frequency response than the voltage amplifier.

This means that transimpedance amplifiers can have a wider bandwidth with a noise performance lower or comparable to high input impedance voltage amplifiers. Regarding the input-referred mean-squared voltage noise of the voltage amplifier, it can be written in a slightly complex form due to the presence of feedback resistances (*R*_1_ and *R*_2_):

(6) $$\begin{array}{c} V_{\mathrm{nVA\_RTI}}^{2}={V_{\mathrm{nph}}^{2}+V}_{\mathrm{ndark}}^{2}+V_{\mathrm{nbg}}^{2}+4\mathrm{kT}R_{S}∆f+V_{n}^{2}+I_{n+}^{2}R_{S}^{2}+I_{n-}^{2}{\left(\frac{R_{1}R_{2}}{R_{1}+R_{2}}\right)}^{2}+\\ 4\mathrm{kT}R_{1}{\left(\frac{R_{2}}{R_{1}+R_{2}}\right)}^{2}∆f+4\mathrm{kT}R_{2}{\left(\frac{R_{1}}{R_{1}+R_{2}}\right)}^{2}∆f. \end{array}$$

In the 4*kTR_s_* (*R*_s_—equivalent source resistance, including also *R*_d_) component, we assumed all the thermal noise generated in the circuity was connected to the *IN*+. Assuming that noise gain is equal to the voltage gain *G* = 1 + *R*_2_/*R*_1_ [34], the output noise RMS can be calculated using

(7) $$V_{\mathrm{nVA\_RTO}}=\sqrt{V_{\mathrm{nVA\_RTI}}^{2}G^{2}}.$$

Regarding the resulting SNR, an important parameter determining the relation between unwanted noise and useful signals, it can be stated that the best performances are usually obtained using high-gain, low-noise TIA with photodiodes characterized by high dynamic resistance and small capacitance. The input-referred noise decreases along with the transimpedance ratio, which is increased by feedback resistance (reducing thermal noise). A low SNR is usually obtained when a TIA operates with a low-resistance photodiode and a low transimpedance gain. Moreover, as shown in the following paragraphs, the voltage configuration is also generally inferior to TIA in terms of performance. Thermal, flicker (1/*f*), and shot noises generated in detector and front-end electronics significantly limit the SNR value. Specific requirements indicate the selected configuration, which balances noise reduction with signal integrity, timing performance, and gain.

## 2. Materials and Methods

Practical noise measurements were provided for different electronic circuits prepared using printed circuit boards (PCBs) and SMD elements. In such a way, the configuration shown in Figure 1a,b and a–c were tested in terms of noise voltage spectral density (VSD) at the output. As a photodiode, the well-known and popular BPW34 from Vishay was used in all cases. This square shape silicon PIN photodiode was characterized by 7.5 mm^2^ of active area, a typical reverse dark current of 2 nA (at *V*_R_ = 10 V), a capacitance of 70 pF (at *f* = 1 MHz and *V*_R_ = 0 V), and a noise equivalent power of 4 × 10^−14^ W/√Hz [35]. In the case of current–voltage converters, the proper frequency compensation adding feedback capacitance (*C_f_*) was provided to obtain gain characteristics as flat as possible while maintaining the largest possible bandwidth with a pulse signal overshot not exceeding a few percentage points. The prepared amplifiers used two types of low-noise voltage feedback (VFB) op-amps, namely AD797A (representing BJT input type) and ADA4625 (representing JFET input type) in SOIC8 packages [36,37]. A feature that distinguishes these types from others is very low voltage noise *V*_n_. The main parameters of these op-amps are summarized in Table 1.

Comparing the parameters of both op-amps, it is evident that AD797A is characterized by lower voltage noise at the cost of higher current noise in comparison with ADA4625-1. Nevertheless, the latter has the benefit of low input bias current in the range of pA, resulting in ultra-low current noise. The differential mode input capacitance is lower in the case of the ADA4625-1 op-amp, whereas common mode capacitance is lower for AD797A. The GBW is more than six times larger in the case of AD797A. A practical circuit was built in such a way so as to minimize the influence of parasitic capacitances that can significantly affect output noise. The photodiode was connected to the op-amp input as closely as possible. The prepared circuit on a two-layer PCB was shielded by mounting it in a metal case with feedthrough connectors to avoid external interferences. A low-noise supply and bias voltage were provided using lead acid batteries. The results were collected for different gain settings. To obtain transimpedances of 10^4^ V/A, 10^5^ V/A, and 10^6^ V/A, the 10 kΩ, 100 kΩ, and 1 MΩ precision (metal film, tolerance 0.5%) feedback resistances were used. The proper feedback capacitances (*C_f_*) were carefully selected. The output noise signal was measured using a precision FFT spectrum analyzer with high input impedance (1 MΩ) and a 16-bit analog-to-digital converter. Moreover, the sampling frequency was selected to obtain a high-resolution spectrum from 10 Hz and avoid aliasing. The spectral density was calculated using the FFT result and its resolution bandwidth (RBW) value. During noise measurements, the photodiode-sensitive area was covered to avoid noise from signals caused by background incident radiation. The rise time used for the bandwidth estimation of each circuit was also measured. For this purpose, a setup with a high-speed semiconductor laser diode (650 nm), a pulse signal generator, and a 12-bit oscilloscope (250 MHz bandwidth) was prepared.

## 3. Results

### 3.1. Configurations with AD797A Op-Amp

**Figure 5:**
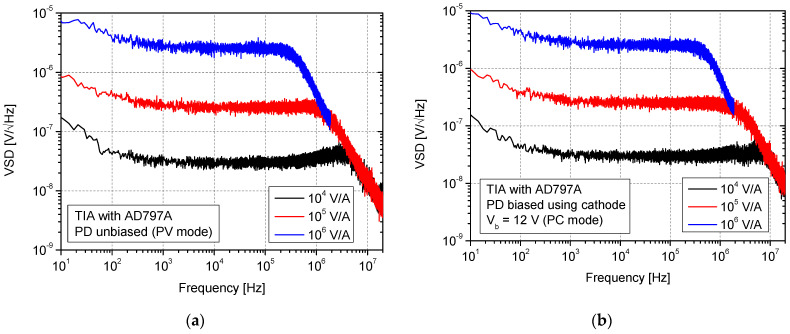
VSD of noise measurement at the output of the TIA with AD797A op-amp without photodiode bias (**a**) and with bias (**b**).

In Figure 5, the results of VSD measurements at the output of the current–voltage converters with the AD797A op-amp without photodiode bias (circuit from Figure 2a) and with a bias applied (circuit from Figure 2b) are shown. The characteristics were collected and presented for different transimpedance gains. These characteristics were measured from 10 Hz up to a frequency exceeding the noise bandwidth of each circuit (determined by “-3 dB” in gain reduction multiplied by a specific coefficient—π/2). The values of these points will be summarized in the form of a table at the end of this section. Nevertheless, they can also be roughly estimated from VSD characteristics.

Analyzing the obtained characteristics, it is evident that the noise observed at the output is increasing along with transimpedance gain. In the case of the unbiased photodiode (at *f* = 10 kHz) for 10^4^ V/A, 10^5^ V/A, and 10^6^ V/A, the values 3 × 10^−8^ V/√Hz, 2.5 × 10^−7^ V/√Hz, and 2.5 × 10^−6^ V/√Hz were measured, respectively. The ten times increase in gain results in an increase in noise, also by about the same factor (one decade). The 1/*f* type of noise was registered up to about 1 kHz. In most cases, the VSD is dominated by the op-amp input current noise source because it is much higher than feedback resistance thermal noise. We also decided to verify our real measurements with SPICE-based calculations. Using such a simulator, we obtained 2.5 × 10^−8^ V/√Hz, 2.2 × 10^−7^ V/√Hz, and 2.2 × 10^−6^ V/√Hz for transimpedance gains of 10^4^ V/A, 10^5^ V/A, and 10^6^ V/A, respectively. As in practice measurements, these results refer to the *f* = 10 kHz and TIA circuit without photodiode bias. The values obtained from simulations are quite close to those obtained experimentally.

**Figure 6:**
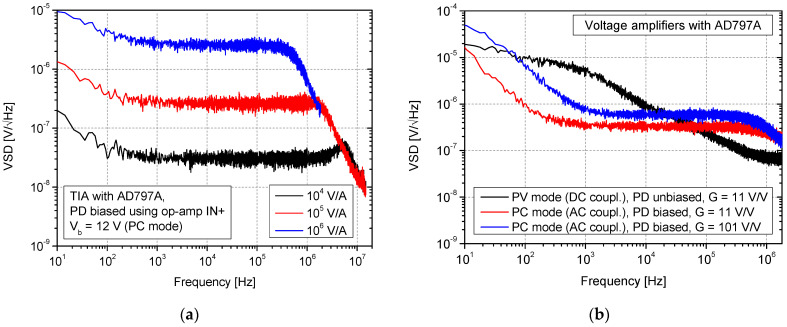
VSD of noise measurement at the output of the TIA with photodiode bias using non-inverting input (**a**) and voltage amplifiers with AD797A prepared for operation with photovoltaic and photoconductive mode (**b**).

Observing VSD behavior in Figure 5a,b, the bandwidth reduction and the increase in transimpedance gain are also clearly noticeable. In the case of 10^4^ V/A and the unbiased photodiode, a bandwidth of at least a few MHz can be estimated. A slight bandwidth increase can be noticed when comparing results for the same transimpedance gain but in different bias situations (no bias vs. bias applied). This is caused by photodiode capacitance reduction (for 12 V of applied reverse bias, *C_d_* is about 15 pF). In general, the use of the bias voltage does not significantly modify the shape of VSD characteristics in the passband. However, it affects the position of the falling slope defining the bandwidth upper limit, so more noise can be expected when referring to the RMS value calculated in the overall (higher) bandwidth. In Figure 6, the results for TIA with photodiode bias provided using op-amp non-inverting input (as in Figure 2c) and voltage configurations (as in Figure 1a,b) are presented. In the case of an AC-coupled configuration (*f_cutoff_* of input high-pass filter set to ≈ 1.5 Hz), two different voltage gains were verified (Figure 6b). A voltage divider circuit with a photodiode and load resistor (*R_L_*) was supplied using a voltage of 12 V (resulting in photodiode reverse bias).

Referring to the obtained VSD results, it can be clearly noticed that, in general, the change in photodiode biasing approach in TIA (by cathode vs. op-amp *IN*+) does not significantly affect noise properties (Figure 5b vs. Figure 6a) in a low-frequency region. The main differences can be noticed in the area of high frequencies and near the low-pass slope. This can be explained by a change in op-amp input capacitance due to the relatively high voltage (12 V) at both input nodes when a bias voltage reference is applied to the *IN*+. BJT input capacitance can depend on the base voltage, especially regarding base emitter capacitance, due to the change in the depletion region width. For this reason, we observed that changing the bias approach in TIA for the same feedback compensation can provide noticeably different results due to the change in this capacitance. Thus, the feedback compensation capacitances (*C_f_*) were corrected after the bias method change.

Regarding the DC-coupled configuration from Figure 1b. (photovoltaic mode) it can be stated that it is characterized by very low bandwidth, even for relatively small voltage gain (Figure 6b). It is mainly caused by significant photodiode and op-amp input capacitances. In this case, the photodiode’s relatively small current efficiency must charge these capacitances without any feedback provided by the op-amp. Moreover, the noise in this bandwidth is, in principle, higher than AC-coupled configurations (circuit from Figure 1a, photoconductive mode). It is caused by the relatively high current noise of the used BJT-input op-amp that, as a result, led to the generation of considerable voltage noise at the high dark resistance of the connected and covered photodiode.

The photoconductive mode circuits are characterized by high 1/*f* noise up to about 1 kHz. It can also be connected with op-amp current 1/*f* noise that increases as the frequency decreases. In the AD797A datasheet, it can be found that for 1 Hz, it is much higher than 10 pA/√Hz. In the case of voltage gain *G* = 11 V/V, the 10 kΩ load resistor was used, whereas in the case of *G* = 101 V/V, *R_L_* = 1 kΩ. For *f* = 10 kHz, higher noise was registered in the case of *G* = 101 V/V (6 × 10^−7^ V/√Hz) compared to *G* = 11 V/V (3.2 × 10^−7^ V/√Hz). At this point, it must be remembered that the thermal voltage noise of the 10 kΩ load resistor is higher than 1 kΩ one. Using the SPICE simulator for the AC-coupled circuit (photoconductive mode) with *G* = 11 V/V, we calculated 2.8 × 10^−7^ V/√Hz at 10 kHz, so the results are similar with about a 14% difference. The bandwidth of these circuits is comparable to the results with a current–voltage converter set to a similar transimpedance gain (10^5^ V/A). A combination of op-amp noise sources and thermal noise from the input circuit forms the white noise of VSD for these configurations.

### 3.2. Configurations with ADA4625 Op-Amp

**Figure 7:**
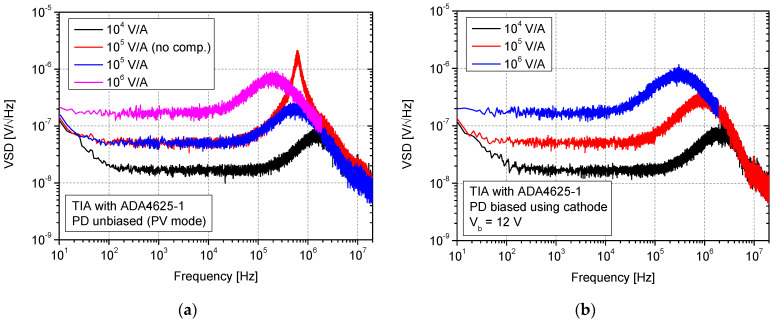
VSD of noise measurement at the output of the current–voltage converters with ADA4625-1 op-amp without photodiode bias (**a**) and with bias (**b**).

In Figure 7, the results of VSD measurement at the output of the TIA with a JFET input ADA4625-1 op-amp are presented. The two situations, without photodiode bias (circuit from Figure 2a) and with bias (circuit from Figure 2b), were also analyzed for different transimpedance gains. In the circuit, intentionally, the same feedback resistances as in the case of AD797A were used for a direct comparison with previous results.

**Figure 8:**
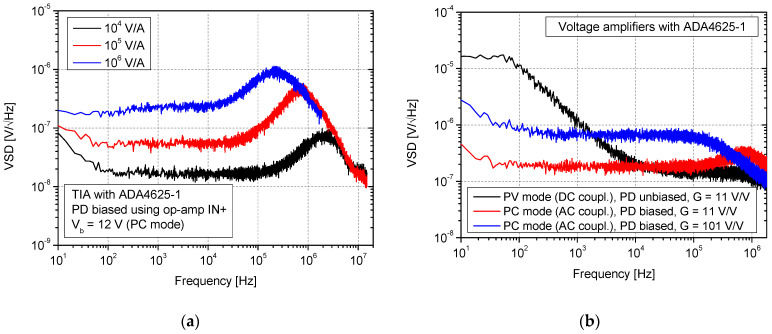
VSD of noise measurement at the output of the TIA with photodiode bias using non-inverting input (**a**) and voltage amplifiers with ADA4625-1 prepared for operation with photovoltaic and photoconductive mode (**b**).

Observing VSD characteristics, it is evident that a significant amount of other noise behavior was obtained in the case of using ADA4625-1 in the TIA circuit. First of all, a characteristic noise bump is visible at high frequencies. Here, it must be highlighted that this bump also occurs after proper amplifier feedback compensation. For this purpose, an example of VSD for 10^5^ V/A without a compensation capacitor (*C_f_*) was also presented in Figure 7a. This specific bump is a well-known phenomenon in TIA circuits [38]. It is caused by the op-amp’s input voltage noise source, which generates current noise on the capacitive reactance component observed at its input [39]. Then, it directly affects TIA noise gain. Due to this fact, all capacitances existing in the circuit should be considered when selecting and designing front-end electronics. The proper calculations can be performed using the SPICE simulator. In the case of AD797A, it was not registered in such a significant form because of the lower value of *V_n_* (Table 1). It can be reduced at the cost of significant bandwidth limitations by using a low-pass filter. Regarding the obtained VSD-specific values, it can also be stated that other behaviors were registered in comparison with AD797A. In general, the noise at 10 kHz, along with transimpedance gain, is not increasing so much as in the case of this op-amp type. Moreover, for each transimpedance gain, a lower value of VSD at *f* = 10 kHz was registered. This confirms the advantages of the JFET input type due to the low noise current in the range of a single fA/√Hz (ADA4625-1). For 10^6^ V/A, we obtained 1.8 × 10^−7^ V/√Hz (10 kHz) vs. 2.5 × 10^−5^ V/√Hz measured with AD797A. For lower transimpedance gains at this frequency, lower values also were measured, at 1.7 × 10^−8^ V/√Hz (10^4^ V/A) and 5.1 × 10^−8^ V/√Hz (10^5^ V/A). Nevertheless, the evident lower bandwidths were noticed for ADA4625-1-based TIAs. For these configurations, the main noise source of white noise is the thermal noise of feedback resistance. Nevertheless, the broadband value will be increased by the specific spectrum bumps caused by *V_n_* and existing capacitances. The differences caused by photodiode biasing are also less visible (similar VSD characteristics were estimated). Simulating using a SPICE-unbiased photodiode in a TIA circuit with ADA4625-1 at *f* = 10 kHz, we obtained 1.5 × 10^−8^ V/√Hz (10^4^ V/A), 4.2 × 10^−8^ V/√Hz (10^5^ V/A), and 1.8 × 10^−7^ V/√Hz (10^6^ V/A). The results of measurements for TIA with photodiode bias provided using an op-amp non-inverting input (Figure 2c) and voltage amplifier configurations are shown in Figure 8.

In this case, the same load resistances (1 kΩ and 10 kΩ) for specific VA gains were used as in the previous circuit with AD797A. Observing the influence of the TIA bias method change (using *IN+*), approximately similar characteristics were obtained, mainly in the low-frequency regions. Nevertheless, higher differences were obtained when comparing the situation with AD797A. This applies in particular to high frequencies where noise is boosted (visible specific bump). Moreover, the FET’s capacitance can significantly depend on the gate voltage, as discussed in [40,41]. Thus, feedback compensating capacitance correction was also needed. Taking into account the results of VSD measured for configurations with voltage amplifiers, it can be stated that the low-frequency noise is formed by the input current noise source of the used op-amp. Due to this fact, significantly lower 1/*f* noise was registered than in AD797A. For the photovoltaic circuit (Figure 1b) at 10 Hz, similar results were obtained for both op-amps (close to the 2 × 10^−5^ V/√Hz), but an also evident lower bandwidth can be noticed in the case of ADA4625-1. Regarding the results of AC-coupled photoconductive configurations, it can be stated that except for the part of the characteristic that refers to the 1/*f* noise, the VSD value is in the same range as AD797A (10^−7^ V/√Hz), but visible lower values were measured in *G* = 11 V/V for ADA4625. This is also caused by the current noise of this op-amp being almost a thousand times lower than the BJT-based one. Verifying the result at *f* = 10 kHz for an AC-coupled (photoconductive) configuration (*G* = 11 V/V) using SPICE, we calculated 1.5 × 10^−7^ V/√Hz (measured 1.8 × 10^−7^ V/√Hz). Commenting on the result for *G* = 101 V/V, higher values were obtained than in AD797A due to *V_n_* being a few times higher and converted to the output by noise gain equal to the *G* factor.

### 3.3. Bandwidth and Broadband Noise Comparison

In the tested circuits, the rise times of the output signal were also measured to calculate approximate bandwidths. The known relation 0.35/*t_r_* was used for this purpose. The optical square-shaped pulses were generated using a semiconductor laser (650 nm) and a signal generator. The output signal was monitored by an oscilloscope with a maximum sample rate of 1.25 GSa/s. The results are summarized in Table 2.

Taking into account data from the table, it is evident that the predicted situation was obtained. The application of PD bias in TIA circuits led to significantly increased bandwidth in each case. As expected, the highest value (close to 9 MHz) was obtained for AD797A and 10^4^ V/A with PD bias. Regarding the PD bias method in TIA, quite similar values were registered (biased cathode vs. bias using *IN+*). Nevertheless, compensation for non-ideal feedback must also be taken into consideration. The slowest circuit is the DC-coupled VA configuration (PD works in photovoltaic mode). Commenting on the bandwidths of VA where PD is operating in photoconductive mode, it can be stated that similar results to the TIA configurations were obtained. In these circuits, the PD current is converted into voltage using *R*_L_ and then amplified by the *G* factor, so the transimpedance can be assumed to be *R_L_G*. Using *R_L_* = 10 kΩ and *G* = 11, the transimpedance is close to 10^5^ V/A.

Based on the obtained VSD characteristics and bandwidths extracted from rise times, we also calculated broadband RMS noise, taking into consideration noise bandwidth (NBW). Because we have different characters (white, 1/*f*) in obtained VSDs, for this purpose, we provided a spectrum integration method in a range from 10 Hz (lower limit of our measurements) to the noise bandwidth upper frequency limit assumed as NBW = 1.57*f*_-3dB_. The results are summarized in Table 3.

The analysis of results is summarized in Table 3. It can be stated that the noise depends on gain and bandwidth in all cases. The highest noise we obtained in the TIA circuit was using AD797A (PD biased) and transimpedance was 10^6^ V/A. The high current noise of this op-amp causes this. In this case, the more-than-ten-times-lower value was calculated for ADA4625-1, but it must be remembered that the bandwidth of the circuit with this op-amp is about 2.5 times smaller. The lowest broadband RMS noise at the output was extracted for TIA with ADA4625-1 (no PD bias). In general, in most cases, the noise oscillates in the range of hundreds of microvolts. Considering both types of used op-amps, the closest noise values were registered for a TIA configuration with biased PD and 10^4^ V/A. Commenting on the influence of the photodiode bias method with TIA, similar results were calculated in most cases. The high differences were obtained for ADA4625-1, where noise VSD bump at high frequencies may have a dominant influence. Therefore, the resulting RMS value is very sensitive to feedback compensation. The results obtained can be used during detector front-end design considering the required performance. Moreover, the direct comparison of different solutions includes the didactic aspect.

Discussing further noise reduction, it can be stated that in the literature, we can find works addressing this issue in both hardware and software methods. Building specific multichannel parallel input stages, cross-correlation-based techniques, or lock-in amplification can be used to reduce noise components [42]. Regarding improvements in hardware circuits, hybrid configurations based on a combination of discrete low-noise transistors with op-amps are also well-known [43,44]. Using such configurations, better noise performance can be obtained in comparison with single-channel semi-op-amp-based circuits.

### 3.4. Example Configuration for Cross-Correlation-Based Noise Reduction

**Figure 9:**
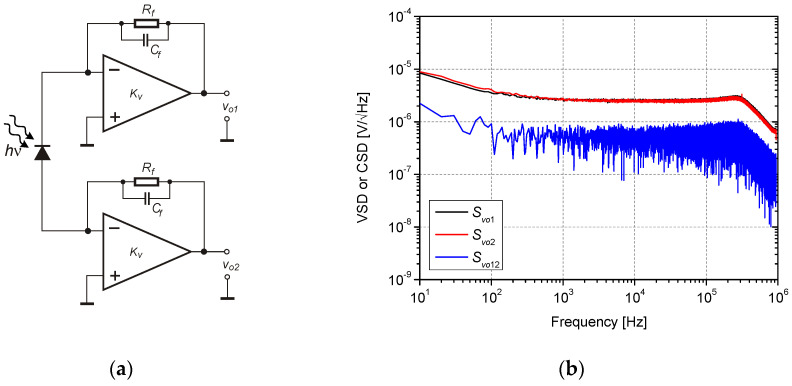
Schematic of conventional differential TIA for cross-correlation method; (**a**) output VSD of each channel and CSD (**b**).

As mentioned before, noise reduction can be obtained using specific prepared circuits and advanced data processing methods. In the case of an application where useful information can be extracted from the spectrum (frequency domain), the conventional configuration of two-channel differential TIA can be used (Figure 9a). It is composed of two identical op-amp-based TIAs operating in series. The photodiode is connected between their inverting inputs. In the case of unbiased operation (photovoltaic mode), both non-inverting inputs are grounded. To obtain photodiode bias (photoconductive mode), the proper low-noise voltage source can be connected to one or both of the non-inverting inputs. The overall input current flows through both TIAs but in opposite directions. Thus, at the outputs (*V_o_*_1_ and *V_o_*_2_), the signal from correlated sources (i.e., *I_nph_*, *I_nd_*) is present with inverted phases (negative correlation). This does not apply to uncorrelated components whose phases in output signals are random. In the presented circuits, the op-amps’ current noise sources generate non-correlated signals at the outputs, whereas voltage noise sources cause the generation of a correlated signal at both outputs. By using two output signals (*V*_o1_ and *V*_o2_) and a cross-correlation algorithm, it is possible to reduce the contribution of op-amps’ current noise and the thermal noise of the feedback resistances [45]. Then, the output-referred cross-spectrum of noise can be described by

(8) $$V_{\mathrm{nRTO\_S}12}=\sqrt{{{\left(I_{\mathrm{nph}}R_{f}\right)}^{2}+\left(I_{\mathrm{ndark}}R_{f}\right)}^{2}+{\left(I_{\mathrm{nbg}}R_{f}\right)}^{2}+V_{n1}^{2}+V_{n2}^{2}}$$

where *V_n_*_1_ and *V_n_*_2_ are regarded as the op-amps’ noise voltage source contributions (assuming *R_d_* >> *R_f_*). In the case of a useful photocurrent signal (*I*_ph_), it will also remain in the computed spectrum because it generates correlated components. In Equation (8), we assumed that existing impedances are dominated by the resistance component.

To present the described noise reduction method in practice, the experiment with the circuit in Figure 9a was also provided. In the prepared circuit, we intentionally used AD797A op-amps and the high-value feedback resistors *R_f_* = 1 MΩ (10^6^ V/A). In the previous section, we elucidated that in such a prepared single TIA configuration, the op-amp current noise (in the range of pA/√Hz) is problematic (dominating). During the experiment, the *v*_o1_ and *v*_o2_ signals were simultaneously sampled and then digitally processed to obtain the VSD of every single channel (*S_vo_*_1_ and *S_vo_*_2_) and CSD (cross-spectral density—*S_vo_*_12_) of both channels. The results are presented in Figure 9b.

Analyzing the spectrums obtained, it is evident that, as expected, the cross-correlation procedure reduced the noise in all frequency ranges, whereas, as indicated before, the op-amp current noise is dominating. The intensity of noise reduction depends, among others, on the number of averages. In our experiment, their number was slightly over 400. The spectra regarded as single channels are basically the same. Comparing VSD to CSD at *f* = 10 kHz, about 2.5 × 10^−6^ V/√Hz vs. 5.4 × 10^−7^ V/√Hz was estimated, so nearly 4.5 times of noise reduction was obtained (20 times in terms of power). Similar results can also be observed in a low-frequency range where 1/*f* noise exists. This circuit can be useful in the case of photodetectors’ noise measurement systems or precision detection systems operating with modulated signals, where the limitation of noise from amplifiers is necessary. The integration of CSD in NBW led to obtaining 4 × 10^−4^ V_rms_, so the four-times-lower RMS value was registered in comparison to a single TIA configuration. The circuit shown in Figure 9a can also be easily converted into an instrumentation amplifier. For this purpose, the two outputs can be connected to the differential amplifier. Such circuits were presented and described in [46,47].

### 3.5. Proposition of Two-Channel Radiation Detector with Differential TIA

**Figure 10:**
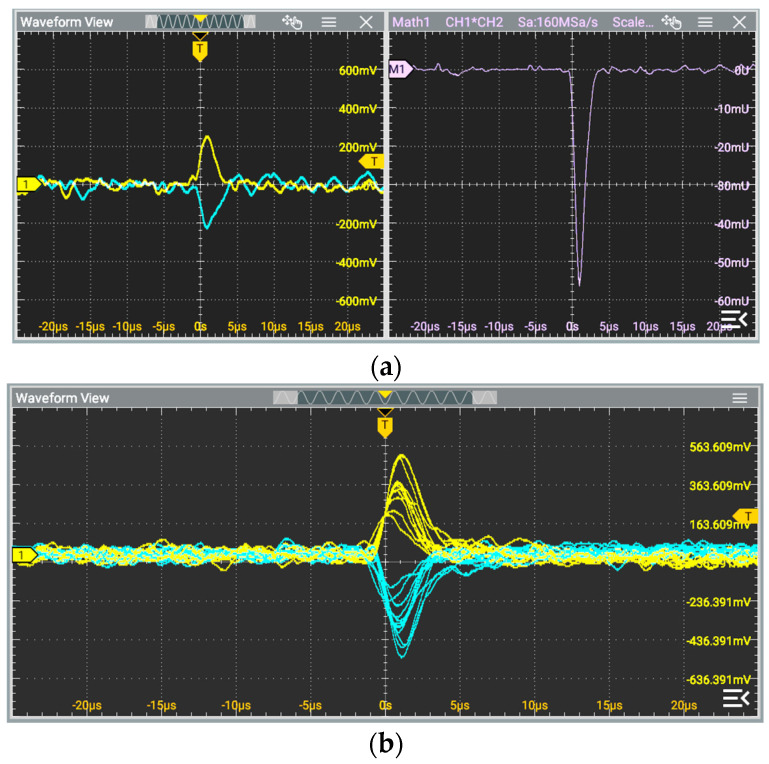
Output signals of two-channel radiation detector with differential TIA and BPW34 (**a**) pulses registered with the scope persistence option enabled (**b**).

As presented in Figure 9 and discussed in the previous section, the noise from some components in differential TIA circuits is uncorrelated. Due to this fact, we decided to verify the operation of this circuit as a simple radiation detector. In general, such a detector should be characterized by high gain, low noise, and enough bandwidth, so in the case of systems requiring high performance (i.e., sensitivity), the design can be a non-trivial task. For the preliminary verification purpose, the circuit shown in Figure 9a was modified based on the results summarized in Table 3. Analyzing these results, it is evident that the FET input op-amp should be used to obtain high gain and low noise. In the construction of differential TIAs, ADA4625-1 op-amps were used with 1 MΩ feedback resistances. Additionally, the output signals (*V_o_*_1_ and *V_o_*_2_) were amplified (10^2^ V/V) by a typical AC-coupled non-inverting configuration using an OPA1641 op-amp. As previously stated, the single BPW34 PIN photodiode was connected to inverting inputs. The other types of photodiodes used in such circuits can be found in [48]. Nevertheless, the proposition of the BPW34 application for such a purpose can also be found in previous works [49,50,51]. The 5 V reverse bias was applied using a TIA non-inverting input. The Americium-241 (^241^Am) radioactive isotope was used for testing purposes. This source emits alpha (α) and gamma (γ) particles. It is commonly used to create ionizing chambers in smoke detectors. The output signals from the prepared circuit were registered and processed by a 12-bit oscilloscope. The results are presented in Figure 10.

Commenting on the obtained results, it can be stated that incident radiation is received at the proposed detector outputs in the form of a few µs correlated pulses with opposite phases (polarity), whereas the noises have random phases (Figure 10a). Due to this fact, the result of simply multiplying these signals is also presented on the right window in this figure. This was obtained using a built-in scope math function. The multiplication led to the gain of time-correlated pulses. This approach (observing two signals) increases the probability of proper pulse detection caused by incident radiation, whereas the risk of detecting a random pulse from noise is reduced. The proper pulse detected in the first channel should be accompanied by an opposite pulse in the second channel. Such a technique can be significantly useful for pulses with relatively small amplitudes compared to noise (i.e., in cases where the comparator level is set close to the noise level). Figure 10b presents several pulses registered with the scope persistence option enabled. The amplitude varies up to a few hundred mV, whereas the noise peak-to-peak value is slightly larger than 100 mV. This noise can be further reduced by increasing feedback resistances in differential TIA, reducing second-stage gain, or applying signal filtering. The prepared output signals can be used for further advanced digital processing and analysis. The use of a differential amplifier can also be considered. In some cases, the proposed detector front-end can be regarded as a more reliable alternative to the single-channel solutions.

## 4. Conclusions

This paper presented the noise measurements of various configurations of photoreceiver input stages. For this purpose, two different types of low-noise op-amps were intentionally used to present results with BJT and JFET input-based types. The popular BPW34 photodiode was used as a detector. Measured characteristics were discussed to explain the dominating noise sources, which introduced further limitations. In constructing the input stage with the BJT input op-amp, it must be remembered that its high current noise can be dominant, especially in the case of high gains. This problem is limited in the case of JFET input op-amps due to low current noise, often in the range of fA/√Hz. Then, the main noise can be generated by feedback resistance (thermal noise). Taking into consideration the configuration type, it is evident that TIA is characterized by the most advantages. Regarding the VA with photodiode working in photoconductive mode, in some cases, similar results can be obtained by selecting proper load resistance and voltage gain. In our experiments, the configuration of VA and PD in photovoltaic mode was characterized by very low bandwidth and high noise. These facts confirm the low usability, which means that this input stage is rarely used in practice. A very interesting phenomenon was observed in the case of different photodiode bias methods in TIA, which can change the frequency response. This is probably caused by the op-amp input capacitance dependence on applied voltage. Additionally, the two-channel cross-correlation TIA was also presented. The experiment with this circuit confirmed that such a configuration is quite useful where noise reduction is needed. Thanks to this, it is possible to limit the influence of uncorrelated noise sources like op-amp current noise, which is particularly troublesome in the case of BJT-based input stage circuits. Based on the results obtained, the two-channel detector with differential FET input TIA was also presented and examined.

## Figures and Tables

**Table 1:** Selected parameters of AD797A and ADA4625-1 op-amps.

Parameter	AD797A	ADA4625-1
Input Type	BJT	JFET
Gain Bandwidth Product (GBW)	typ. 110 MHz	typ. 16 MHz
Input Noise Voltage Density (*v*_n_)	typ. 1.7 nV/√Hz at *f* = 10 Hz	typ. 5.5 nV/√Hz at *f* = 10 Hz
	typ. 0.9 nV/√Hz at *f* = 1 kHz	typ. 3.3 nV/√Hz at *f* = 1 kHz
Input Noise Current Density (*i*_n_)	typ. 2 pA/√Hz at *f* = 1 kHz	typ. 4.5 fA/√Hz at *f* = 1 kHz
Input Bias Current (*I_b_*)	typ. ± 250 nA	typ. ± 15 pA
Offset Voltage (*V*_os_)	typ. ± 25 µV	typ. ± 15 µV
Input Capacitance (diff. mode) (*C_indm_*)	typ. 20 pF	typ. 8.6 pF
Input Capacitance (com. mode) (*C_incm_*)	typ. 5 pF	typ. 11.3 pF

**Table 2:** The tested configurations’ bandwidth (*f*_-*3dB*_) was calculated using rise time.

	**TIA 10^4^ V/A (no PD bias)**	**TIA 10^5^ V/A (no PD bias)**	**TIA 10^6^ V/A (no PD bias)**
**ADA4625-1**	1.89 MHz	461 kHz	164 kHz
**AD797A**	3.89 MHz	1.37 MHz	357 kHz
	**TIA 10^4^ V/A** **(PD biased by cathode)**	**TIA 10^5^ V/A** **(PD biased by cathode)**	**TIA 10^6^ V/A** **(PD biased by cathode)**
**ADA4625-1**	3.18 MHz	875 kHz	200 kHz
**AD797A**	8.75 MHz	2.00 MHz	515 kHz
	**TIA 10^4^ V/A** **(PD biased by IN+)**	**TIA 10^5^ V/A** **(PD biased by IN+)**	**TIA 10^6^ V/A** **(PD biased by IN+)**
**ADA4625-1**	3.24 MHz	814 kHz	201 kHz
**AD797A**	8.33 MHz	1.94 MHz	519 kHz
	**VA (DC-coupl.)** **G = 11 V/V**	**VA (AC-coupl.)** **G = 11 V/V**	**VA (AC-coupl.)** **G = 101 V/V**
**ADA4625-1**	1.40 kHz	714 kHz	200 kHz
**AD797A**	1.49 kHz	1.19 MHz	761 kHz

**Table 3:** Broadband RMS noise was calculated for the frequency range from 10 Hz to 1.57*f*_-3dB_.

	**TIA 10^4^ V/A (no PD bias)**	**TIA 10^5^ V/A (no PD bias)**	**TIA 10^6^ V/A (no PD bias)**
**ADA4625-1**	9.72 × 10^−5^ V_RMS_	1.39 × 10^−4^ V_RMS_	2.87 × 10^−4^ V_RMS_
**AD797A**	9.50 × 10^−5^ V_RMS_	3.37 × 10^−4^ V_RMS_	1.60 × 10^−3^ V_RMS_
	**TIA 10^4^ V/A** **(PD biased by cathode)**	**TIA 10^5^ V/A** **(PD biased by cathode)**	**TIA 10^6^ V/A** **(PD biased by cathode)**
**ADA4625-1**	1.34 × 10^−4^ V_RMS_	2.78 × 10^−4^ V_RMS_	3.46 × 10^−4^ V_RMS_
**AD797A**	1.15 × 10^−4^ V_RMS_	3.84 × 10^−4^ V_RMS_	1.90 × 10^−3^ V_RMS_
	**TIA 10^4^ V/A** **(PD biased by IN+)**	**TIA 10^5^ V/A** **(PD biased by IN+)**	**TIA 10^6^ V/A** **(PD biased by IN+)**
**ADA4625-1**	1.28 × 10^−4^ V_RMS_	3.93 × 10^−4^ V_RMS_	4.23 × 10^−4^ V_RMS_
**AD797A**	1.20 × 10^−4^ V_RMS_	4.15 × 10^−4^ V_RMS_	1.90 × 10^−3^ V_RMS_
	**VA (DC-coupl., PV mode)** **G = 11 V/V**	**VA (AC-coupl., PV mode)** **G = 11 V/V**	**VA (AC-coupl., PC mode)** **G = 101 V/V**
**ADA4625-1**	1.80 × 10^−4^ V_RMS_	2.90 × 10^−4^ V_RMS_	3.10 × 10^−4^ V_RMS_
**AD797A**	2.78 × 10^−4^ V_RMS_	3.60 × 10^−4^ V_RMS_	5.40 × 10^−4^ V_RMS_

## Data Availability

The original contributions presented in the study are included in the article; further inquiries can be directed to the corresponding author.
